# Lessons learnt from the impact of COVID-19 on arthroplasty services in Hong Kong: how to prepare for the next pandemic?

**DOI:** 10.1186/s42836-021-00093-5

**Published:** 2021-09-06

**Authors:** Lok Sze Lee, Ping Keung Chan, Wing Chiu Fung, Amy Cheung, Vincent Wai Kwan Chan, Man Hong Cheung, Henry Fu, Chun Hoi Yan, Kwong Yuen Chiu

**Affiliations:** 1 Department of Orthopaedics and Traumatology, The University of Hong Kong, Hong Kong, China; 2 Department of Orthopaedics and Traumatology, Queen Mary Hospital, Hong Kong, China

**Keywords:** Arthroplasty, Replacement, Total knee arthroplasty, Total hip arthroplasty, COVID-19

## Abstract

**Background:**

Arthroplasty services worldwide have been significantly disrupted by the pandemic of coronavirus disease 2019 (COVID-19). This retrospective comparative study aimed to characterize its impact on arthroplasty services in Hong Kong.

**Methods:**

From January 1 to June 30, 2020, the patients of “COVID-19 cohort” underwent elective total hip or knee replacement in Hong Kong public hospitals. The cohort was compared to the “control cohort” during the same period in 2019. Data analysis was performed to compare the two cohorts’ numbers of operations, hospital admission, orthopaedic clinic attendances, and waiting time.

**Results:**

A total of 33,111 patient episodes were analyzed. During the study period, the elective arthroplasty operations and hospitalizations decreased by 53 and 54%, respectively (*P* < 0.05). Reductions were most drastic from February to April, with surgical volume declining by 86% (*P* < 0.05). The primary arthroplasty operations decreased by 91% (*P* < 0.05), while the revision operations remained similar. Nevertheless, 14 public hospitals continued performing elective arthroplasty for patients with semi-urgent indications, including infection, progressive bone loss, prosthesis loosening, dislocation or mechanical failure of arthroplasty, and tumor. At the institution with the highest arthroplasty surgical volume, infection (28%) was the primary reason for surgery, followed by prosthesis loosening (22%) and progressive bone loss (17%). The orthopaedic clinic attendances also decreased by 20% (*P* < 0.05). Increases were observed in waiting time and the total number of patients on the waiting list for elective arthroplasty.

**Conclusions:**

Despite the challenges, public hospitals in Hong Kong managed to continue providing elective arthroplasty services for high-priority patients. Arthroplasty prioritization, infection control measures, and post-pandemic service planning can enhance hospital preparedness to mitigate the impact of current and future pandemics.

## Background

In order to help contain the coronavirus disease 2019 (COVID-19) outbreak, many hospitals worldwide have reduced elective operations to redeploy the resources for handling the challenges of the pandemic. However, since most arthroplasties are elective procedures, joint replacement services have been significantly disrupted.

The orthopaedic operations were subjected to one of the greatest relative declines in the normal medical activity in both the United Kingdom (UK) National Health Service and the United States (US) [1, 2]. Although there are less abundant data for Asian regions, a major Singapore institution reported a 74% reduction in arthroplasties compared to the pre-pandemic levels [3], while a major orthopaedic department in Japan noted over 80 and 50% reductions in hip and knee arthroplasties respectively [4]. Similar patterns were observed in mainland China, Malaysia, and South Korea [5, 6].

Hong Kong reported its first confirmed COVID-19 case on January 23, 2020. The initial outbreak had a relatively flat epidemic curve. This was followed by a second and a third wave beginning in March and July 2020, respectively, both related to the imported cases. Hong Kong is currently experiencing its fourth wave, with a cumulative total of 10,710 confirmed cases (including 10,022 discharged and 188 deaths) since February 10, 2021. The Hong Kong Government’s response level was raised to “Emergency”–the highest tier–on January 25, 2020, with subsequent public health measures including school suspension, restricting public gatherings, and postponing non-urgent hospital procedures [7]. The reduction in elective operations has resulted in considerable disruptions in the local arthroplasty services. In Hong Kong, 30% of the elderly aged over 65 years are diagnosed with osteoarthritis, and the knee and hip joints are mostly affected [8, 9]. Given the disease prevalence and associated morbidity and disability, disruptions in arthroplasty services have important implications on patient outcomes and future joint replacement services when hospitals return to normal surgical schedules.

Therefore, this retrospective comparative study aimed to review the impact of COVID-19 on the arthroplasty services in Hong Kong’s public health care system, to inform strategies to mitigate the impact of current and future pandemics.

## Methods

The ethics approval was granted by the Institutional Review Board of the University of Hong Kong/Hospital Authority Hong Kong West Cluster (reference number: UW 20–594).

This retrospective cohort study was conducted in Hong Kong, which has a dual-track public and private healthcare system for a population of 7.5 million [10]. The public sector provides over 90% of inpatient services in the region and handles all suspected and confirmed COVID-19 cases [11]. In our study, we retrospectively analyzed the data collected from all 43 public hospitals and 122 outpatient clinics. Diagnoses and procedures were classified according to the International Classification of Diseases, 9th Edition, Clinical Modification (ICD-9-CM).

The “COVID-19 cohort” of patients who underwent elective joint replacement surgery (including primary and revision total hip and knee arthroplasty) from January 1 to June 30, 2020 was compared to the “control cohort” selected during the same period in 2019. We performed data reconciliation of aggregate and individual data, followed by statistical analyses using Welch’s *t*-test. We assessed whether the differences in continuous outcomes between the two cohorts were statistically significant at the 5% significance level. The primary outcomes included the number of operations, hospital admission, and orthopaedic clinic attendances. The secondary outcomes included the nature of operations performed, indications for arthroplasty at our institution, and waiting time for the elective arthroplasty.

## Results

We analyzed 33,111 patient episodes, including 3080 operations, 3031 hospitalizations, and 27,000 orthopaedic clinic attendances.

### Arthroplasty operations

**Fig. 1:**
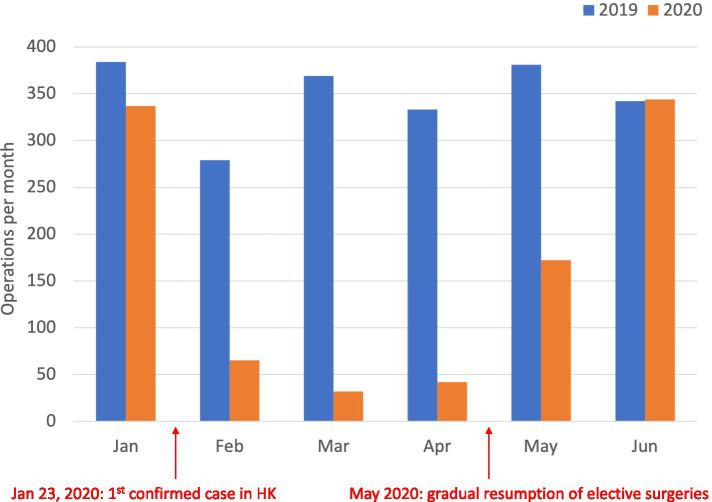
Total surgical volume of elective hip and knee arthroplasties in Hong Kong (HK) public hospitals

Regarding surgical volume and hospitalizations from January 1, 2020 to June 30, 2020, the elective joint replacement operations decreased by 53% (from 348 ± 39.6 to 165.3 ± 144.6 per month; *P* = 0.026), compared to the control cohort (Fig. 1), while the hospital admissions fell by 54% (from 345 ± 38.3 to 160.2 ± 145.6 per month; *P* = 0.03). The reductions were most drastic from February to April. This period followed the Hospital Authority directives of reprioritizing non-urgent and non-essential services under the government’s “emergency” response level raised on January 25, 2020 [7]. The surgical volume and hospital admissions for elective joint replacement declined by 86% (*P* = 0.004) and 88% (*P* = 0.003) respectively (Table 1). This period followed Hong Kong’s first COVID-19 case confirmed on January 23, 2020 and preceded the gradual resumption of elective services in the public hospitals around May 2020. During this time, reductions in surgical volume varied across operative categories. Primary arthroplasty operations decreased by 91% (from 315.3 ± 45.9 to 28 ± 23.1 per month; *P* = 0.003), while the number of revision operations remained similar (*P* = 0.21). The ratio of hip to knee arthroplasties increased from 1:5.3 to 1:1.8.

**Table 1:** Differences in elective arthroplasty services from February to April

	COVID-19 Cohort (2020)	Control Cohort (2019)	Change	*P* Value
	Monthly Mean ± SD	Monthly Mean ± SD		
Operations				
Total	46.3 ± 16.9	327 ± 45.3	-85.8%	0.004
Revision joint replacement				
Revision hip arthroplasty	10 ± 4.6	7 ± 2	42.9%	0.382
Revision knee arthroplasty	8.3 ± 4.2	4.7 ± 2.5	78.6%	0.276
Primary joint replacement				
Total hip arthroplasty	6.7 ± 2.1	45 ± 6.6	-85.2%	0.006
Total knee arthroplasty	21.3 ± 21.0	270.3 ± 43.5	-92.1%	0.003
Hospital admissions	40 ± 19.7	325 ± 44.3	-87.7%	0.003
Orthopaedic clinic attendances	1652.3 ± 143.5	2336.3 ± 200.1	-29.3%	0.011

Despite massive cutbacks from February to April 2020, 14 public hospitals continued to perform elective joint replacement operations. Queen Mary Hospital accounted for the majority (26%) of cases, and detailed analysis revealed that infection (28%) was the primary reason for surgery, followed by implant loosening (22%) and bone loss (17%).

### Outpatient clinic attendances

From January 1, 2020 to June 30, 2020, the COVID-19 cohort had 20% fewer orthopaedic outpatient clinic attendances than the control cohort (reduction from 2,495 ± 243.5 to 2,005 ± 426.5 per month; *P* = 0.04). The increases were observed in both waiting time and total number of patients on the waiting list for elective joint replacement surgery. For example, the recent aggregate data collected from the Hospital Authority showed a 14% growth in the arthroplasty waitlist (from 26,547 to 30,342) from March to December 2020 [12], despite an overall 8% (from 106,472 to 98,153) decline in the past 12-month new-case bookings (from the interval between April 1, 2019 and March 31, 2020 to the interval between January 1, 2020 to December 30, 2020) at the orthopaedic outpatient clinics [13]. It reflected a net increase in total case load despite fewer new cases at outpatient clinics.

## Discussion

COVID-19 profoundly impacts the arthroplasty services. Many institutions worldwide have postponed non-emergent operations to reduce hospital traffic, conserve personal protective equipment, and enable manpower deployment to COVID-19 frontline services. Likewise, orthopaedic services in Hong Kong have been significantly reduced, particularly for joint replacement and ligamentous reconstruction procedures, as the majority are elective operations [14].

Apart from the Hospital Authority policies [7], patient health-seeking behavior also contributes to those reductions due to COVID-19 risk. Our institution’s arthroplasty prioritization and infection control measures are summarized in Table 2. Specifically, the elective joint replacement services are maintained primarily for high-priority patients with increased morbidity and likelihood of necessitating more complicated reconstruction procedures if the operations are delayed [15]. The key semi-urgent indications for proceeding with elective arthroplasty included (1) infection; (2) progressive bone loss; (3) loosening, dislocation, or mechanical failure of arthroplasty; and (4) tumor (Table 2). It is in line with the international guidelines proposed by the professional bodies regarding the provision of clinical services during the outbreak, such as the American College of Surgeons guidelines [16] (Table 3) and the clinical guide for orthopaedic surgical prioritisation established at the request of the UK National Health Service [17] (Table 4). For example, infection is one of the essential indications (wound drainage, fever, concern for infection with prior joint replacement) for surgery in the American College of Surgeons’ guidelines, and the UK guidelines list “infection” and “septic arthritis (natural or prosthetic joint)” as priority 1a indications, *i*.*e*., emergency arthroplasty should be performed within 24 h. Progressive bone loss is a priority 2 indication (*i*.*e*., a suggested timeframe < 1 month) in the UK guidelines as “destructive bone lesion with risk of fracture (*e*.*g*., giant cell tumor)”. Loosening, dislocation, or mechanical failure of arthroplasty are in accordance with American College of Surgeons’ essential indications of “knee dislocation”, “prior hip or knee replacement with acute pain exacerbation”, while the UK guidelines classify “dislocated joints” as priority 1a and “revision surgery for loosening without impending fracture or recurrent joint instability” as priority 3 (*i*.*e*., arthroplasty should be performed within 3 months). Tumor is listed in the UK guidelines as priority 2 for “solitary metastasis”.

**Table 2:** Summary of infection control and arthroplasty service prioritization at our institution

	Guidelines and measures
Patient screening	Inpatient admission screening • SARS-CoV-2 RT-PCR test using deep throat saliva self-collected by patient in the presence of a HEPA filter unit Outpatient screening (*e*.*g*., preoperative clinic appointments) • Patients are screened for symptoms and signs of COVID-19 using a health declaration form
Visiting arrangement	• All ward visitations are suspended except for compassionate visit of inpatients for exceptional situations on a case-by-case basis • Visitors are screened for symptoms and signs of COVID-19 using a health declaration form • Visitors are required to wear full personal protective equipment, including face shield, N95 respirator, isolation gown and disposable gloves
Arthroplasty prioritisation	Proceed with elective arthroplasty for semi-urgent indications, such as: • Joint infection • Loosening, dislocation or mechanical failure of arthroplasty • Bone loss • Tumor Preoperative screening • SARS-CoV-2 RT-PCR test using deep throat saliva self-collected by patient in the presence of a HEPA filter unit

**Table 3:** US arthroplasty scheduling recommendations, adapted from the American College of Surgeons [16]

	Phase II (curtail elective practice)		Phase III (eliminate elective practice)	
	Proceed	Postpone	Proceed	Postpone
Acute knee or hip pain	✓			✓
Chronic knee or hip pain		✓		✓
Inability to weight bear	✓		✓^a^	
Knee or hip dislocation	✓		✓	
Concern for periprosthetic joint infection	✓		✓	
Acute pain exacerbation with prior joint replacement	✓		✓	

^a^Only for acute inability to weight bear

**Table 4:** National Health Service arthroplasty scheduling recommendations, adapted from the Federation of Specialty Surgical Associations (UK) [17]

**Priority**	**Surgery timeframe**	**Orthopaedics & traumatology examples**
1a	< 24 h	• Infection: *e*.*g*., septic arthritis (natural or prosthetic joint)
		• Dislocated joints
1b	< 72 h	• Unstable articular fractures that will result in severe disability without operative fixation
2	< 1 months	• Destructive bone lesion with risk of fracture (*e*.*g*., giant cell tumour)
		• Solitary metastasis
		• Arthroplasty – any site where delay will prejudice outcome
3	< 3 months	• Revision surgery for loosening without impending fracture, or recurrent joint instability
4	> 3 months	• Arthroplasty/arthrodesis – not otherwise specified

The rationale underlying those recommendations takes into consideration multiple parameters, including the immediate risk, the long-term impact of the disease, and the projected future severity [18]. Since the COVID-19 situation and resource availability vary by region in Asia, individual localities will benefit from developing specific recommendations on arthroplasty prioritization, to balance between tackling COVID-19 and minimizing the service disruption.

Infection control measures are based on the local Hospital Authority guidelines [19]. All inpatients at our institution (*e*.*g*., patients admitted for joint replacement surgery) are screened for COVID-19 at admission. To minimize the risk of nosocomial transmission, we performed a SARS-CoV-2 RT-PCR test for all inpatients using a deep throat saliva sample, and the screening scheme has now been expanded to patients attending day services [19]. Similar to the practices in Japan [4] and South Korea [20], such pre-arthroplasty screening allows an elective arthroplasty to be continued throughout the outbreak. Another strategy recommended by the UK National Health Service is to require patients and their household members to self-isolate for 14 days before admission for elective operations [21]. It potentially reduces the risk of transmission in case of insufficient testing capacity.

In addition, patients attending day services at our pre-operative assessment clinic are also screened for the signs and symptoms of COVID-19 and TOCC (travel, occupation, contact, and cluster) history via a patient declaration form. In Hong Kong, although visiting wards has been suspended in light of the current wave of outbreak, local public hospitals currently allow the compassionate visit of inpatients for exceptional situations on a case-by-case basis [19]. The visitors are similarly screened for the signs and symptoms of COVID-19 before they are allowed entry to the wards, and full personal protective equipment is required during the visit.

The rapid return to fully functioning pre-pandemic baseline surgical volume by June 2020 reflects adequate facility readiness to resume normal elective joint replacement services upon dampening of the second wave of outbreak. It was supported by the Hospital Authority directives, such as encouraging healthcare departments to increase weekend clinical service hours under the Special Honorarium Scheme from late May to September 2020 to take care of previously postponed cases due to COVID-19 restrictions.

Hong Kong subsequently faced its third and fourth waves of COVID-19 infection beginning in July and November 2020, respectively. The experience of managing previous waves of the outbreak, together with the increased availability of infection control equipment worldwide, results in enhanced outbreak preparedness and response measures in public hospitals. Together, the extended efforts of our institution contribute to the timely detection of occult cases and limiting the spread of COVID-19, while allowing elective arthroplasty to proceed for semi-urgent patients (Table 2).

The growing waiting list for elective joint replacement reflects a greater impact of the pandemic on the surgical volume than that on the outpatient clinic attendances. The pent-up demand for elective joint replacement procedures is anticipated to pose significant challenges even after services recover to pre-pandemic full capacity. It is a looming global public health crisis with two major implications. First, a rising number of osteoarthritis patients are facing delayed surgical management. Knee osteoarthritis is the primary reason for disability in walking, housekeeping, and stair-climbing among non‐institutionalized individuals aged 50 years or above [22]. A postponed arthroplasty adversely affects patient outcomes due to significant deterioration in morbidity during the delay and worse health-related quality of life outcomes following late arthroplasty [23–26]. Second, the operation backlog represents a major future burden on the health care system. Estimates showed that if countries increased surgical capacity by 20% following the pandemic, a median of 45 weeks will be required to work through the surgical backlog due to COVID-19 disruptions [27]. A May 2020 analysis of elective orthopaedic surgery under the COVID-19 outbreak found that in the optimistic scenario, the US will have a cumulative backlog of over 1 million surgical cases 2 years following the resumption of elective surgeries, and it may take as many as 16 months to address 90% of the surgical backlog [28].

Hong Kong has been fortunate to have rapidly returned to pre-pandemic service provision levels upon dampening of previous outbreak waves. Nevertheless, planning for the anticipated backlog remains critical. Strategies for arthroplasty service planning during different stages of the pandemic are summarized in Table 5. In addition to guidelines on the resumption of surgical services published by professional organizations [29, 30], an evidence-based three-phase return pathway for elective orthopaedic operations has been proposed by orthopaedic surgeons at the Croydon University Hospital London and South West London Elective Orthopaedic Centre [31], which suggests stratifying patients into risk groups, so as to begin service resumption for low-risk patients with maximum anticipated benefit in quality of life (*e*.*g*., patients suitable for day case arthroplasty) and gradually expanding to all orthopaedic cases. Developing regional post-pandemic arthroplasty resumption guidance for hospitals will not only facilitate the return to normal clinical services but also avoid creating inequalities in waiting time among patients in different hospital catchment areas.

**Table 5:** Strategies for arthroplasty service planning during the next pandemic

Pre-pandemic	• Build a consensus among stakeholders for prioritization of arthroplasty services, including inpatient, outpatient and operation, during different degrees of severity of a pandemic
	• Establish guidelines for infection control measures for patients and health care workers during the pandemic
	• Establish guidelines for operating on a confirmed infected case during the pandemic
	• Set up telemedicine infrastructure for preoperative education, outpatient consultation and follow-up, and telerehabilitation
	• Set up ERAS services for arthroplasty procedures
During the pandemic	• Adjust clinical services according to the severity of the pandemic
	• Increase the capacity for supporting ERAS services in arthroplasty to shorten hospital stay and reduce the burden on inpatient care
	• Provide telemedicine consultations for pre-operative education and postoperative follow-up
	• Provide telerehabilitation to maintain mobility and knee function; ensure access to drug-refill clinic for patients on waiting list for arthroplasty
	• Provide telerehabilitation for postoperative rehabilitation after arthroplasty
	• Develop a post-pandemic arthroplasty resumption plan for the anticipated backlog
Post-pandemic	• Prepare manpower and hospital capacity for the post-pandemic increase in clinical service (*e*.*g*., extend operating room schedules)
	• Utilize orthopaedic block times for arthroplasty procedures
	• Enhance mental health support for healthcare workers to cope with the increase in workload during the post-pandemic phase

Increasing application of Enhanced Recovery After Surgery (ERAS) for arthroplasty procedures has delivered promising results in Singapore and Hong Kong in terms of reduced hospital length of stay and comparable risk of complications [32–34]. The use of telemedicine for outpatient clinic consultations has also been discussed, particularly for postoperative follow-up appointments of suitable patients, in order to reduce hospital traffic while reaching patients who hesitate to visit hospitals due to concerns regarding the outbreak [35]. Other potential strategies reported in the literature included extending operating room schedules to increase surgical capacity [28]; shifting to same-day arthroplasty for carefully selected patients at the major arthroplasty centers [36]; and utilizing orthopaedic block times to boost efficiency during the dedicated operative time for arthroplasty to clear the backlog more quickly and efficiently [37]. Furthermore, enhancing mental health support for healthcare workers may help alleviate the anxiety associated with the surge of patients during the post-outbreak phase [38].

With rapid globalization and rising interconnectedness between humans and natural environments, there is an increasing concern on the emergence of new pandemics [39]. The local and global experience during the COVID-19 pandemic can inform the arthroplasty service planning and post-pandemic recovery to enhance hospital and community preparedness for current and future pandemics.

## Conclusions

Despite the drastic service reduction, public hospitals in Hong Kong continued providing elective joint replacement services for high-priority patients. Hong Kong’s experience in the past few months can inform the service planning for post-pandemic recovery and in anticipation of future crises such as a new wave of COVID-19 pandemic.

## Data Availability

The datasets used and/or analyzed during the current study are available from the corresponding author on reasonable request.

## Abbreviations

COVID-19: Coronavirus disease 2019
HK: Hong Kong
UK: United Kingdom
US: United States
